# Potentials of Chitosan-Based Delivery Systems in Wound Therapy: Bioadhesion Study

**DOI:** 10.3390/jfb3010037

**Published:** 2012-01-06

**Authors:** Julia Hurler, Nataša Škalko-Basnet

**Affiliations:** Drug Transport and Delivery Research Group, Department of Pharmacy, University of Tromsø, Universitetsveien 57, Tromsø N-9037, Norway; E-Mail: Natasa.Skalko-Basnet@uit.no

**Keywords:** chitosan, Carbopol, hydrogel, texture analysis, wound therapy, bioadhesion

## Abstract

Chitosan is currently proposed to be one of the most promising polymers in wound dressing development. Our research focuses on its potential as a vehicle for nano-delivery systems destined for burn therapy. One of the most important features of wound dressing is its bioadhesion to the wounded site. We compared the bioadhesive properties of chitosan with those of Carbopol, a synthetic origin polymer. Chitosan-based hydrogels of different molecular weights were first analyzed by texture analysis for gel cohesiveness, adhesiveness and hardness. *In vitro* release studies showed no difference in release of model antimicrobial drug from the different hydrogel formulations. Bioadhesion tests were performed on pig ear skin and the detachment force, necessary to remove the die from the skin, and the amount of remaining formulation on the skin were determined. Although no significant difference regarding detachment force could be seen between Carbopol-based and chitosan-based formulations, almost double the amount of chitosan formulation remained on the skin as compared to Carbopol formulations. The findings confirmed the great potential of chitosan-based delivery systems in advanced wound therapy. Moreover, results suggest that formulation retention on the *ex vivo* skin samples could provide deeper insight on formulation bioadhesiveness than the determination of detachment force.

## 1. Introduction

Wound therapy represents one of the challenging areas in drug product development, as in the United States alone more than 6.5 million patients are affected annually and the costs of treatment is estimated to be US$ 25 billion per year [1]. Wound infections, particularly of burn wounds, are the most serious complications related to burn injuries [2]. Up to 1% of population requires medical treatment each year due to burn injuries [3].

The consensus within the wound therapy field recommends that modern wound dressings should preserve a humid environment and create a protective barrier against both mechanical stress and secondary infections. The dressing should enable absorption of wound exudate and potential microorganisms, as well as be safe and nonirritant. Other important properties of a wound dressing are its acceptability to the patient and cost per unit [4].

Chitosan is a polysaccharide obtained through partial deacetylation of chitin, which is often derived from shells and crustaceans [5]. Chitosan is biodegradable, with hemostatic, bacteriostatic and wound healing properties, and is, therefore, used in a wide range of biomedical applications [6]. Based on those facts, we focused on the development of chitosan-based delivery systems for wound therapy, particularly burn therapy. Since many drugs and active ingredients with potentials for wound treatment are poorly water soluble, liposomes were used as a drug carrier system serving as drug solubilizer. Liposomes are known to reduce skin irritation by sustaining the release of drugs and by hydration of the epidermis [7]. Due to the fact that the application of wound dressing might be painful for the patient, sustained release is a desirable property of the formulation, enabling the reduction in need for dressing change.

Successful wound treatment is dependent on many factors. Besides the effectiveness of the active ingredient, textural properties of the formulation and drug release pattern, it is also of great importance for how long will the formulation remain on the wounded skin. Wound exudates complicate the adhesion of formulation at the site of application due to high water content. Good bioadhesiveness can assure prolonged retention time for applied drug formulation to guarantee an effective local drug concentration and the expected pharmacological response. Consequently, an increase in the bioadhesiveness results in an improvement in the therapeutic outcome. Bioadhesion studies enable a better overview of the formulation’s retention time and its applicability, indicating the success of the therapy.

## 2. Experimental Section

### 2.1. Materials

Carbopol^®^ Ultrez 10 was a product of Noveon (Cleveland, OH, USA). Low Mw chitosan (Brookfield viscosity 20.000 cps, degree of deacetylation (DDof 92), medium Mw chitosan (Brookfield viscosity 200.000 cps, DD of 82) and high Mw chitosan (Brookfield viscosity 800.000 cps, DD of 77) were purchased from Sigma Aldrich Chemistry (St. Luis, MO, USA). Chloramphenicol was purchased from the Norsk Medisinal Depot (Oslo, Norway). Triethylamine was obtained from Merck Schuchardt (Hohenbrunn, Germany) and glycerol was purchased from Merck KGaA (Darmstadt, Germany). Lipoid S100 was a generous gift of Lipoid GmbH (Ludwigshafen, Germany). Triglycerides (medium chain) were obtained from Fagron GmbH&Co KG, Barsbüttel, Germany. All other chemicals used in experiments were of analytical grade.

The skin used in the bioadhesion experiments was derived from the inner part of pig ear. The samples were kindly provided from the local animal department (Department of Comparative Medicine, University of Tromsø, Tromsø, Norway). The skin was shaved and thoroughly rinsed prior to experiments.

### 2.2. Methods

#### 2.2.1. Preparation of Carbopol Hydrogels

Carbopol^®^ Ultrez 10 hydrogels were prepared by the modified method of Fresno *et al.* [8]. In short, the amount of Carbopol powder (to prepare 0.5% and 1.0% w/w mixtures, respectively) was dispersed in distilled water. For deprotonation of the acid groups in polymer matrix, triethylamine (defined quantity) was added to adjust the pH of hydrogels to be between 6 and 7. The gels were left to swell for at least 24 h at room temperature before further experiments.

#### 2.2.2. Preparation of Chitosan Hydrogels

Chitosan hydrogels were prepared as previously described [9]. Low, medium and high molecular weight chitosans were dissolved in a blend of diluted acidic acid (2.5%, w/w) and glycerol (10%, w/w), respectively. The mixtures were hand-stirred for 10 min and put in an ultrasonic bath for additional 30 min to remove entrapped air bubbles. The gels were allowed to swell for 48 h at room temperature prior to further handling.

#### 2.2.3 Preparation and Characterization of Liposomes

The conventional dry film method [10] was used for preparation of liposomes. Lipoid S100 (200 mg) was dissolved in approximately 20 mL of methanol. The solvent was completely removed on a rotary vacuum evaporator (Büchi R-124, Büchi Labortechnik, Flawil, Switzerland). The remaining lipid film was rehydrated with 10 mL of distilled water and hand shaken for at least 5 min.

For *in vitro* release studies chloramphenicol as a model drug was incorporated into liposomes. Chloramphenicol (20 mg) was dissolved together with Lipoid S100 (200 mg) in methanol. Liposomes containing chloramphenicol were prepared in the same manner as described for empty liposomes.

The size of liposomes (approximately 1 µm, polydispersity index 0.637) was determined by dynamic light scattering (NICOMP submicron particle sizer, model 370, Nicomp Particle Sizing system, Langhorne, PA, USA). For determination of entrapment efficiency of chloramphenicol, the liposomal dispersion was ultracentrifuged for 25 min at 10 °C and 32,000 rpm (Beckmann-L8-70M ultracentrifuge, Beckmann instruments Inc., Palo Alto, CA, USA).

Pellet, containing chloramphenicol loaded liposomes, was dissolved in methanol and, together with supernatant (unentrapped drug) analyzed by HPLC (Waters separation module 2695 and waters 2487 UV-spectrometer detector, Waters Milford, MA, USA; with XTerra^TM^ RP_18_ 5 µm (3.9 × 150 mm) W01671T 004 column, Waters Milford, MA, USA). The mobile phase consisted of methanol:filtrated H_2_O:acetic acid (glacial) in ratio 55:45:0.1. Temperature of column and samples was maintained at 35 ± 2 °C during the chromatographic separation. The flow rate was 1 mL/min and a running time was 5 min. UV detection wave-length was set at 270 nm [11]. 

#### 2.2.4. Preparation of Liposomal Hydrogels

Liposomal dispersion (10%, w/w) was carefully incorporated into the hydrogel by hand-stirring [12]. Random samples of liposomal hydrogels were evaluated and uniformity of liposomes distribution within the hydrogel network confirmed (data are not shown).

#### 2.2.5. Texture Analysis

All chitosan hydrogels were characterized by texture analysis according to Hurler *et al.* [9]. In brief, a Texture Analyzer TA.XT Plus (Stable Micro Systems Ltd., Surrey, UK) was used for backwards extrusion measurements. A disc (40 mm diameter) was pushed at a speed of 4 mm/s for a distance of 10 mm into the hydrogel (60 g) and redrawn. The gel hardness was determined and texture properties such as cohesiveness, adhesiveness were calculated.

The hardness was represented by the maximal force that is achieved during the downwards movement of the disc. Cohesiveness was the work that is required to compress the disc into the gel, whereas adhesiveness was the adequate work in the upwards movement of the disc, representing a measure of the ability of the formulation to adhere on the disc. 

The measurements were performed in triplicates, whereof each sample was measured five times.

#### 2.2.6. *In-Vitro* Release Studies

**Figure 1:**
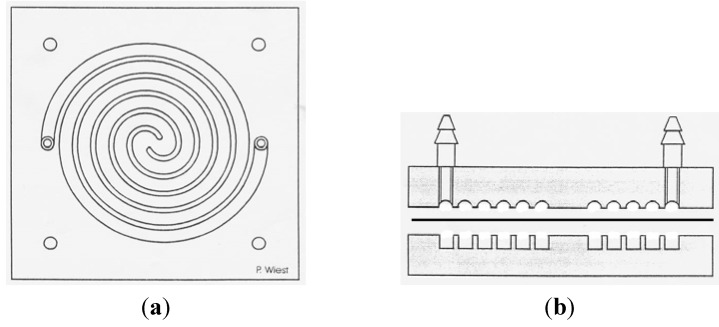
Spiral release system: (**a**) Top view of the donor site; (**b**) Side view of the release chamber: the upper part shows the receiver site; the lower part shows the donor site.

The release studies were performed in a spiral release chamber, with 25 g of formulation in the donor part of the release system. The system was designed and manufactured at University of Freiburg, Germany to be applied in the release studies of semi-solid dosage forms such as creams, gels, *etc*. (Figure 1). 

The receiver phase consisted of 50 mL of medium chain triglycerides. A polyamide membrane (Sartorius AG, Göttingen, Germany) separated the two phases. Samples were drawn at 0.25, 0.5, 1, 2, 3, 4, 6, 8, 12 and 24 h time period [11]. The amount of released chloramphenicol was quantified by HPLC (see Section 2.2.3).

The release profiles were fitted according to the following equation: M_t_/M_∞_ = k × t^1/2^. M_t_/M_∞_ is the ratio of the absolute cumulative amounts of drug released at time t to the absolute amount of drug incorporated with the system at time t = 0, *k* is a release constant represented by the slope of the linear regression analysis of the relation between the fraction of released chloramphenicol and the square root of time [13].

The release studies were performed in triplicates.

The samples (hydrogels in our case) were evenly filled into the donor part of the release cell and covered with the membrane. Subsequently, the receiver part was inserted on the top and fastened. The release cells were connected to a receiver reservoir by tubes, allowing for the receiver phase to be pumped through the system (Ismatec IPC, Ismatec SA, Glattbrugg, Switzerland).

#### 2.2.7. Bioadhesion Test

Bioadhesion test was performed by two different methods. The first method was based on modified method of Keegan *et al.* [14], originally developed for formulations destined for buccal therapy. A mucoadhesion rig for a Texture Analyzer TA.XT Plus (Stable Micro Systems Ltd., Surrey, UK) was used to test bioadhesion. The formulation to be tested (150 µL corresponding to 140 ± 10 µg) was applied by the help of a one-mL syringe onto the die, which was part of the mucoadhesion test rig. A skin slice was clamped onto the membrane holder and rinsed thoroughly with ethanol (96%, v/v) prior to experiment. The die was pinched for exactly 10 s with a pressure of 25 g onto the skin slice. The die was redrawn from the skin at a speed of 0.1 mm/s until the gel slipped. The detachment force was recorded.

The second method used in bioadhesion test was focused on the determination of the exact amount of formulation that remained on the skin slice, as we expected that this parameter will provide deeper insight on the formulation bioadhesiveness. The amount of the formulation retained on the skin slice mimics more closely the actual conditions encountered by wound dressing in wound therapy. For that purpose, the die was weighed before and after the testing. All tests were performed in triplicates and each formulation was tested five times. In between the measurements, the skin slice was rinsed with ethanol.

## 3. Results and Discussion

### 3.1. Texture Analysis

Successful treatment of wounds or burns depends not only on the activity of the topically applied drug but also on the properties of the vehicle in applied formulation. Texture analysis provides deeper insight on the vehicle properties and enables correlation to applicability of the formulation. Texture properties such as gel cohesiveness, adhesiveness and hardness can be measured in a straight forward measure [9]. The results of measurements for different chitosan-based hydrogels are presented in Table 1.

**Table 1:** Texture properties of chitosan hydrogels of different molecular weights chitosans.

Hydrogel	Cohesiveness (g*s)	Adhesiveness (g*s)	Hardness (g)
LMW chitosan, 6.0%	516.0 ± 11.0	−323.0 ± 9.0	223.9 ± 7.8
MMW chitosan, 3.5%	386.5 ± 31.6	−299.1 ± 20.4	168.8 ± 11.0
HMW chitosan, 2.5%	570.8 ± 5.1	−426.4 ± 1.5	250.7 ± 4.6

During characterization of chitosans of various molecular weights and same concentration of polymer, we realized that viscosity of formulations was very much different (data not shown) implicating difference in texture properties as well. The choice of chitosan concentrations used in the study was based on expected similarities in textural properties. Nevertheless, MMW chitosan (3.5%, w/w) showed to be the weakest in respect to gel hardness of the tested gels, whereas HMW chitosan (2.5%, w/w) was shown to be the strongest (Table 1). 

We reported previously [9] that the direct comparison of results obtained by texture analysis of hydrogels made of different polymers (e.g., chitosan and Carbopol) is not reliable due to the fact that the experimental set up needs to be adjusted to the gel properties. Gel hardness, cohesiveness and adhesiveness for Carbopol-based hydrogels was found to be higher than for chitosan-based hydrogels [9]. For example 0.5% (w/w) Carbopol hydrogels showed cohesiveness in range of several thousand g*s, whereas all chitosan hydrogels (Table 1) showed values in the range of several hundred g*s. The most similarities were observed in gel hardness, for which 6% LMW chitosan gel exhibited similar hardness to low concentrations of Carbopol gels [9]. The focus of current work was on full characterization of chitosan gels (Table 1). All three types of chitosan hydrogels appeared to have satisfactory cohesiveness, adhesiveness and hardness to be applied to the skin and wounded area. Based on the findings that HMW chitosan gels exhibited similar textural properties at much lower polymer concentration as compared to MMW and LMW chitosan gels, they were selected for further evaluation.

### 3.2. *In-Vitro* Release Studies

Drug release from topical formulations is affecting the efficiency of topical therapies to a great extent. Chloramphenicol, a model antimicrobial drug, which can be used in topical wound treatment [15], was incorporated into liposomes and its release properties from both Carbopol and chitosan-based delivery systems tested. Medium chain triglycerides (MCT) were chosen as the receiver phase, as it is known that MCT have been shown to have similar properties as the stratum corneum [16].

**Figure 2:**
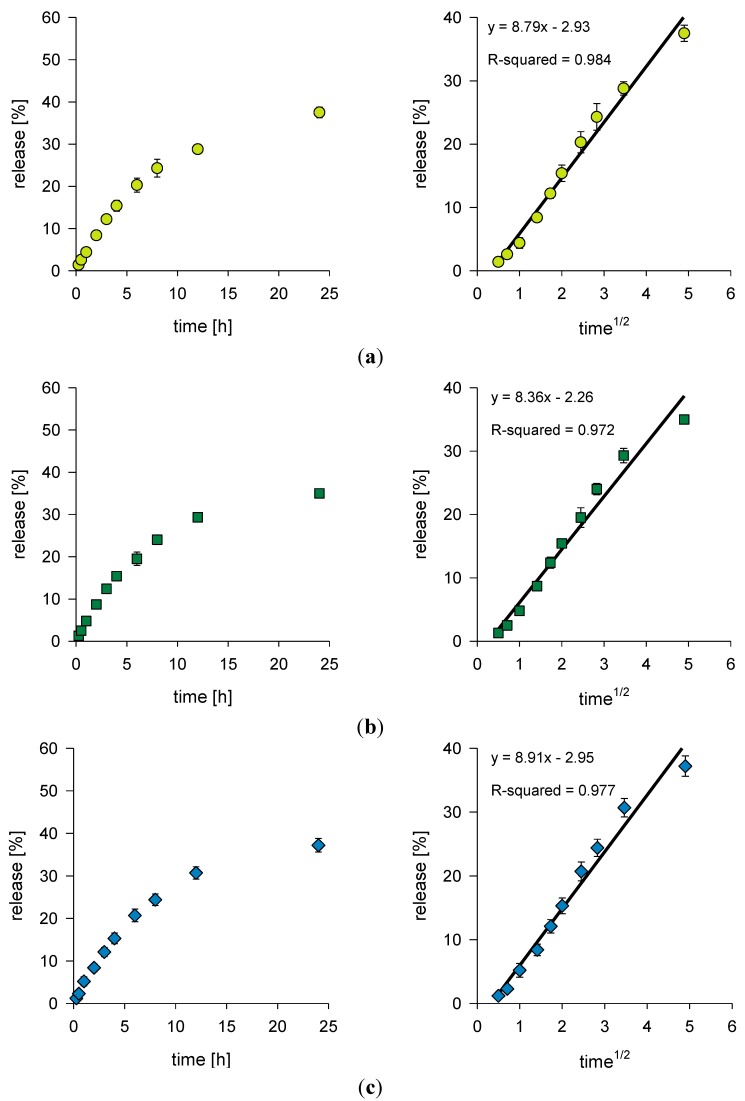
Release profiles of chloramphenicol: Percent release relative to the square root of time. (**a**) HMW (2.5%, w/w) chitosan-based liposomal hydrogel; (**b**) Carbopol (0.5%, w/w) liposomal hydrogel; (**c**) Carbopol (1.0%, w/w) liposomal hydrogel.

Drug release profiles from several tested formulations are shown in Figure 2(a–c). After a 24 h time period, no significant difference in drug release could be seen between the three different formulations. Although the chitosan formulation had released the highest amount of chloramphenicol (37.5% of the incorporated chloramphenicol was released in total), Carbopol hydrogels (both concentrations) showed a very similar release pattern. It could be explained by potential of HMW chitosan and Carbopol Ultrez 10 hydrogels to have a similar microviscosity, although the bulk viscosity might be different, as suggested by Gabrijelcic and Sentjurc for xantan hydrogels [17]. Al-Khamis and group claimed that drug release from gel is controlled by two factors, namely the thermodynamic activity of the drug and the microviscosity of the gel [18]. Ji *et al.* confirmed these findings through the determination of protein release from hydroxypropyl methylcellulose gels [19]. Kristl *et al.* [20] suggested that in the microenvironment of chitosan high molecular weight gels, the drug release is strongly affected by the degree of deacetylation of chitosan. 

Another indicator for the similar microviscosity of HMW chitosan (2.5%) and Carbopol Ultrez 10 gels (0.5 and 1.0%) is the similarity in the values of the release constant *k* (8.79, and 8.36 and 8.91, respectively), which are reflecting structural and geometric characteristics of the hydrogel [21].

Chloramphenicol release is expected to be fully diffusion controlled as the cumulative percentage of drug released is proportional to the square-root of time. The regression lines show a good fit with R^2^ ≥ 0.97. Higuchi reported this kind of relation between release and square-root of time for suspension-ointments originally [22]. The findings in our study are in accordance to the results of a release study for salicylates from Carbopol 940 hydrogels of different concentrations, which showed also a diffusion controlled release [8]. 

### 3.3. Bioadhesion

#### 3.3.1. Measurement of Detachment Force

**Figure 3:**
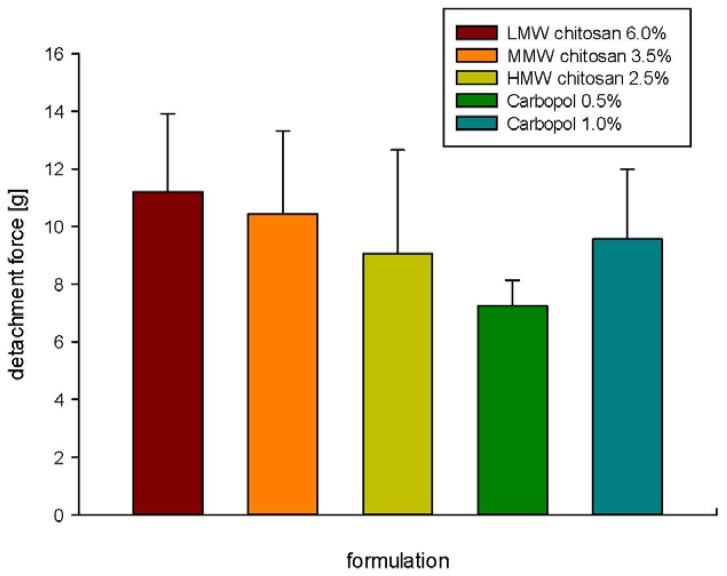
Bioadhesiveness of different hydrogel formulations determined as detachment forces.

Adhesion of topical formulations on wounded skin is of a great importance for the efficiency of topical wound treatment. In Figure 3 the results of the detachment force measurements of all tested formulations are presented. Detachment force is defined as the force needed to overcome the adhesive bond between formulation and skin, when redrawing the die from the skin. In addition, it can be correlated to the cohesiveness of the formulation and shear [23]. 

Qi and Tester [24] tested bioadhesion of tablets made of compressed Carbopol and chitosan. They found that chitosan exhibited less bioadhesiveness than Carbopol. However, we could not see a significant difference between tested formulations made of two different polymers. On the contrary, a higher force was needed to detach the die from chitosan-based formulations compared to Carbopol-based formulations, though it was not significant.

Although the chitosan formulations showed significant difference in their textural properties, namely gel cohesiveness, adhesiveness and hardness, no significant difference could be seen in the detachment force values.

#### 3.3.2. Measurement of the Amount of the Retained Formulation on Skin

Most of the bioadhesion studies reported till date were performed with solid dosage forms for buccal [24,25] or vaginal [26] applications. Jones *et al.* studied bioadhesion of semisolid polymer formulation destined for buccal application [27]. To the best of our knowledge, there are no reported studies dealing with semisolid formulations intended for application onto the skin. Especially wounded skin remains to be a challenge in topical therapy due to the presence of wound exudate. Therefore it is crucial not only to determine the detachment force as a measure of bioadhesion but also to determine the actual amount of formulation that remains onto the skin.

**Figure 4:**
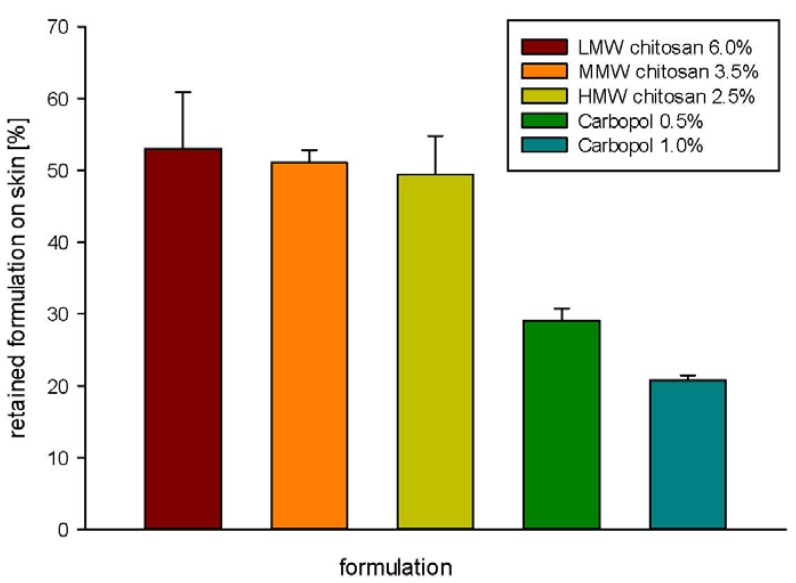
Bioadhesiveness of different hydrogel formulations determined as the amount of retaining formulation on the skin.

Determination of the remaining amount of the formulation (bioadhesion testing) revealed the significant differences between Carbopol-based formulations and chitosan-based formulations (Figure 4). Significantly larger (p < 0.05, *t* test) amount of chitosan formulation remained on the skin after the removal of the die, as compared to Carbopol formulation. Carbopol (1.0%, w/w) formulation showed the lowest retention value. This can be attributed to the lower cohesion of Carbopol 0.5%, as previously reported by us [9]. Although the adhesive capacity of carbopol hydrogels was extensively studied by Blanco-Fuente *et al.* [28], the differences in parameters used in measuring bioadhesion in their study (adhesion work measured on tensile tester) and our study do not permit direct comparison. 

All chitosan formulations showed similar amount of retained hydrogel on the animal skin, indicating that bioadhesion is a complex interaction of many different factors, including gel cohesiveness and adhesiveness. In the case of chitosan, also the degree of deacetylation might play an important role [20].

These findings confirmed that chitosan has a great potential in advanced wound therapy. By developing chitosan-based hydrogels, we can achieve prolonged release of liposomally incorporated drug and assure good bioadhesiveness of the whole formulation onto the wounded area. Although Carbopol is known for its good bioadhesion [24], chitosan showed to be better in retaining on the skin at similar measuring conditions (detachment force values).

## 4. Conclusions

Chitosan has a great potential as a dressing for advanced wound therapy. Chitosan possesses not only hemostatic, biodegradable, bacteriostatic and non-toxic properties, but the present study confirmed its good bioadhesiveness and potential to provide, in combination with liposomes, sustained drug release, which is highly beneficial for wound treatment. Moreover, an expanded method of measuring bioadhesion was developed, including not only the detachment force that is necessary to remove a die from the skin, but also the possibility to determine the actual amount of formulation that remains on the skin.
